# Certain polynomials and related topological indices for the series of benzenoid graphs

**DOI:** 10.1038/s41598-019-45721-y

**Published:** 2019-06-24

**Authors:** Muhammad Nadeem, Awais Yousaf, Hanan Alolaiyan, Abdul Razaq

**Affiliations:** 1 Department of Mathematics, The Islamia University of Bahawalpur, Bahawalpur, 63100 Pakistan; 2 Department of Mathematics, King Saud University, Riyadh, 11451 Saudi Arabia; 3 Department of Mathematics, University of Education Lahore, Jauharabad Campus, Jauharabad, 41200 Pakistan

**Keywords:** Chemistry, Pure mathematics

## Abstract

A topological index of a molecular structure is a numerical quantity that differentiates between a base molecular structure and its branching pattern and helps in understanding the physical, chemical and biological properties of molecular structures. In this article, we quantify four counting polynomials and their related topological indices for the series of a concealed non-Kekulean benzenoid graph. Moreover, we device a new method to calculate the PI and Sd indices with the help of Theta and Omega polynomials.

## Introduction

Graph theory has numerous applications in modern chemistry. In chemical graph theory, the vertices and edges respectively represent the atoms and bonds of a molecular structure. To predict the chemical structure using numerical quantity (i.e., topological indices) graph theory plays a vital role. Topological indices have many applications in theoretical chemistry, especially in QSPR/QSAR research. Numerous researchers have conducted studies on topological indices for different graph families; these indices have important chemical significance in the fields of chemical graph theory, molecular topology, and mathematical chemistry. Diudea was the first chemist to consider the subject of computing the topological indices of nanostructures^1–3^. A small particle of an object of intermediate size (between the microscopic and molecular structures of the object) is called a nanoparticle of that object. Nanoparticles are products derived through engineering at the molecular scale.

Let *G* (*V*, *E*) be a connected graph with a vertex set *V* and an edge set *E*. For any two vertices *v*_1_ and *v*_2_ in *G*, the distance between *v*_1_ and *v*_2_ is denoted by *d* (*v*_1_, *v*_2_)—the shortest path between *v*_1_, and *v*_2_. If *e* is the edge formed by joining *v*_1_ and *v*_2_, and *f* is an edge formed by joining *v*_3_ and *v*_4_, then *e* = *v*_1_*v*_2_ and *f* = *v*_3_*v*_4_ are called codistance edges if *d* (*v*_1_, *v*_2_) = *d* (*v*_3_, *v*_4_) and *d* (*v*_1_, *v*_2_) = *d* (*v*_3_, *v*_4_) = *d* (*v*_1_, *v*_4_) + 1 = *d* (*v*_3_, *v*_2_) + 1 and is denoted by ‘*e co f*’. Here, the corelation is symmetric and reflexive but not transitive. Let *C* (*e*) = {*f* ∈ *E* (*G*); *f co e*}: if the ‘*co*’ relation is transitive, then the set *C* (*e*) is called the orthogonal cut and denoted by *co* of *G*. The set of opposite edges that lie along the same face or the same ring, eventually forming a strip of adjacent faces or rings, is called an opposite edge strip and denoted by ‘*ops*’. This concept is also termed a quasi-orthogonal cut, denoted by ‘*qoc*’. Here, the *co* distance edges are defined within the entire graph *G*, while ‘*ops*’ are defined in the same face or ring. By *m* (*G*, *c*), we mean the number of strips of length *c*. In this paper, we constructed four polynomials: Omega, Sadhana, Theta and PI. Counting polynomials are those polynomials whose exponent is the extent of a property partition and whose coefficients are the multiplicity of the corresponding partition. We also calculated the topological indices related to these polynomials and formulae. Each counting polynomial represents interesting topological properties of the molecular graph. These polynomials are constructed on the basis of quasi-orthogonal cut edge strips for the series of concealed non-Kekulean benzenoid graphs. The counting polynomials and matching polynomials are useful for topologically describing bipartite structures as well as for counting some single-number descriptors (i.e., the topological indices). The Omega and Theta polynomials count equidistant edges of the graph, while the Sadhana and PI polynomials count nonequidistant edges. Various results related to counting polynomials and topological indices can be found in^1–14^.

The Omega polynomial of a graph *G* (*V*, *E*) is denoted by*ω* (*G*, *x*); more information can be found in^15,16^. The Omega polynomial is defined as $\omega (G,x)=\sum _{c}\,m(G,c){x}^{c}s$, where *c* is the length of the strip, and ‘*m* (*G*, *c*)’ is the number of strips of length *c*. The Sadhana polynomial was defined as, $Sd(G,x)=\sum _{c}\,m(G,c){x}^{|E(G)|-c}$ in^17^, where |*E* (*G*)| is the size of the graph G. The Theta polynomial of graph *G*is denoted by ‘*θ* (*G*, *x*)’ and defined as, $\theta (G,x)=\sum _{c}\,m(G,c)c{x}^{c}$ in^18^. The PI polynomial is defined in^4^ as *PI* (*G*, *x*) =$\sum _{c}\,m(G,c)c{x}^{|E(G)|-c}$.

## Series of Concealed Non-Kekulean Benzenoid Graph

**Figure 1:**
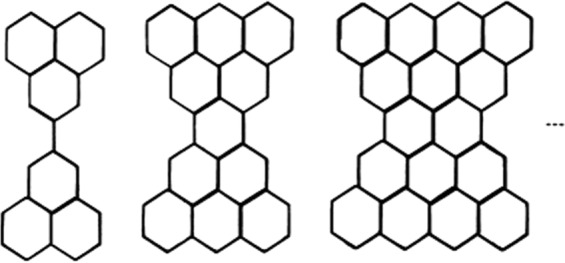
Series of concealed non-Kekulean benzenoid graph.

The Kekulean and non-Kekulean structures in benzenoids have important properties from a chemical point of view. It is known that most benzenoids with different numbers of starred and unstarred vertices have no Kekulean structure; these possess color excess and are referred to non-Kekulean benzenoids. In contrast, benzenoids with equal numbers of starred and unstarred vertices necessarily possess Kekulean structures. According to Gutman^19^, equal numbers of starred and unstarred vertices is a necessary and sufficient condition for a benzenoid structure to be Kekulean. However, it is not true that non-Kekulean with equal numbers of starred and unstarred vertices were detected and later identified as concealed non-Kekulean benzenoids. The total number of edges in the series of concealed non-Kekulean benzenoid graphs shown in Figure 1.1 is 17*n* + 14, where *n* is the number of connected edges in the middle of the graph. It has been demonstrated that exactly eight systems of this category exist. If we eliminate the edge cut, which consists of the connected edges, then the graph is decomposed into two parts. Such a structure is called the Kekulean structure of the benzenoid graph. In the present work, we use the concealed non-Kekulean benzenoid graph shown in Fig. 1 ^19–23^.

The series of concealed non-Kekulean benzenoid graphs in Fig. 1 has six quasi-orthogonal cuts (i.e., *S*_*i*_ *i* = 1, 2 … 6) of different lengths. The lengths of the cuts (qoc) and the number of cuts in the series of concealed non-Kekulean benzenoid graphs are *n*, *n* + 1, *n* + 2, 2, 3, and 6 and 1, 2, 2, 4, 4, and 2(*n* − 1), respectively.

### Theorem 1.1

The Omega and Theta polynomials for the series of concealed non-Kekulean benzenoid graph are as follows:

$$\omega (G,\,x)={x}^{n}+2\sum _{i=1}^{2}{x}^{n+i}+4\sum _{i=1}^{2}{x}^{1+i}+2(n-1){x}^{6},$$

and

$${\rm{\Theta }}(G,\,x)=n{x}^{n}+2\sum _{i=1}^{2}(n+i){x}^{n+i}+4\sum _{i=1}^{2}(1+i){x}^{1+i}+2(n-1){x}^{6}.$$

### Proof

: To calculate the Omega and Theta polynomials of the concealed non-Kekulean benzenoid graphs shown in Figure 1.1, we need to find both the quasi-orthogonal cuts (qoc) and the number of quasi orthogonal cuts of each type. Let *S*_*i*_, *where i* = 1, 2 … 6 be six ‘qoc’ in a concealed non-Kekulean benzenoid graph. The lengths and cardinalities of these quasi-orthogonal cuts (i.e*., S*_*i*_, *where i* = 1, 2 … 6) are *n*, *n* + 1, *n* + 2, 2, 3, *and* 6 and 1, 2, 2, 4, 4, *and* 2(*n* − 1), respectively. Because the Omega polynomial is defined as, *ω* (*G*, *x*) =$\sum _{c}m(G,\,c){x}^{c}$, where c is the length of the cut and *m* (*G*, *c*) represents the number of quasi-orthogonal cuts of length c. Hence, the Omega polynomial calculated from the qocs of a concealed non-Kekulean benzenoid graph is:

$$\omega (G,\,x)={x}^{n}+2\sum _{i=1}^{2}{x}^{n+i}+4\sum _{i=1}^{2}{x}^{1+i}+2(n-1){x}^{6}.$$

Also, by the definition of the Theta polynomial, Θ (*G*, *x*) =$\sum _{c}\,m(G,c)c{x}^{c}$. Hence, the Theta polynomial calculated from the cuts of a concealed non-Kekulean benzenoid graph becomes

$${\rm{\Theta }}(G,\,x)=n{x}^{n}+2\sum _{i=1}^{2}\,(n+i){x}^{n+i}+4\sum _{i=1}^{2}\,(1+i){x}^{1+i}+12(n-1){x}^{6}.$$

### **Theorem 1.2**

The Sadhana and PI polynomials for the series of concealed non-Kekulean benzenoid graphs shown in Figure 1.1, are as follows:

$$sd(G,\,x)={x}^{16n+14}+2{x}^{16n+13}+2{x}^{16n+12}+4{x}^{17n+12}+4{x}^{17n+11}+2(n-1){x}^{17n+8},$$

and

$$\pi (G,\,x)=n{x}^{16n+14}+2(n+1){x}^{16n+13}+2(n+2){x}^{16n+12}+8{x}^{17n+12}+12{x}^{17n+11}+12(n-1){x}^{17n+8}.\,$$

### Proof

: In the series of concealed non-Kekulean benzenoid graphs, the total number of edges is |*E* (*G*)| = 17*n* + 14, and there are six strips (of qocs) of different lengths, namely, *S*_*i*,_*i* = 1, 2 … 6. The lengths of these strips are *n*, *n* + 1, *n* + 2, 2, 3, and 6, respectively. The cardinality of the length of *S*_*i*,_*i* = 1, 2 … 6 is1, 2, 2, 4, 4, and 2(*n* − 1). From the definition of the Sadhana polynomial, *sd* (*G*, *x*) =$\sum _{c}m(G,\,c){x}^{|E(G)|-c}$. Therefore, the Sadhana polynomial constructed from the cuts of a concealed non-Kekulean benzenoid graph is:

$$sd(G,\,x)={x}^{16n+14}+2{x}^{16n+13}+2{x}^{16n+12}+4{x}^{17n+12}+4{x}^{17n+11}+2(n-1){x}^{17n+8}$$

Additionally, the *π* Polynomial is defined as *π* (*G*, *x*) =$\sum _{c}m(G,\,c)c.{x}^{|E(G)|-c}$. By using the lengths of the strips and the number of strips in a concealed non-Kekulean benzenoid graph, the π polynomial becomes;

$$\pi (G,\,x)=n{x}^{16n+14}+2(n+1){x}^{16n+13}+2(n+2){x}^{16n+12}+8{x}^{17n+12}+12{x}^{17n+11}+12(n-1){x}^{17n+8}\,$$

Table 1. lists the first five polynomials of all four counting polynomials for concealed non-Kekulean benzenoid graphs.

**Table 1:** First five counting polynomials for the series of concealed non-Kekulean benzenoid graphs.

*n*	Ω(*G*, *x*)	Θ (*G*, *x*)	π(*G*, *x*)	Sd(*G*, *x*)
1	6*x*^3^ + 6*x*^2^ + *x*	18*x*^3^ + 12*x*^2^ + *x*	*x*^30^ + 12*x*^29^ + 18*x*^28^	*x*^30^ + 6*x*^29^ + 6*x*^28^
2	2*x*^6^ + 2*x*^4^ + 6*x*^3^ + 5*x*^2^	12*x*^6^ + 8*x*^4^ + 18*x*^3^ + 10*x*^2^	10*x*^46^ + 18*x*^45^ + 8*x*^44^ + 12*x*^42^	5*x*^46^ + 6*x*^45^ + 2*x*^44^ + 2*x*^42^
3	4*x*^6^ + 2*x*^5^ + 2*x*^4^ + 5*x*^3^ + 4*x*^2^	24*x*^6^ + 10*x*^5^ + 8*x*^4^ + 15*x*^3^ + 8*x*^2^	8*x*^63^ + 15*x*^62^ + 8*x*^61^ + 10*x*^60^ + 24*x*^59^	4*x*^63^ + 5*x*^62^ + 2*x*^61^ + 2*x*^60^ + 4*x*^59^
4	8*x*^6^ + 2*x*^5^ + *x*^4^ + 4*x*^3^ + 4*x*^2^	48*x*^6^ + 10*x*^5^ + 4*x*^4^ + 12*x*^3^ + 8*x*^2^	8*x*^80^ + 12*x*^79^ + 4*x*^78^ + 10*x*^77^ + 48*x*^76^	4*x*^80^ + 4*x*^79^ + *x*^78^ + 2*x*^77^ + 8*x*^76^
5	2*x*^7^ + 10*x*^6^ + *x*^5^ + 4*x*^3^ + 4*x*^2^	14*x*^7^ + 60*x*^6^ + 5*x*^5^ + 12*x*^3^ + 8*x*^2^	8*x*^97^ + 12*x*^96^ + 60*x*^93^ + 5*x*^94^ + 14*x*^92^	4*x*^97^ + 4*x*^96^ + *x*^94^ + 10*x*^93^ + 2*x*^92^

## Topological Indices for the Series of Concealed Non-Kekulean Benzenoid Graph

The numerical value of the first derivatives of these counting polynomials at *x* = 1 yields the interesting properties of the molecular graph. These values are called the topological indices of the graphs. At *x* = 1, the value of the first derivative of the Omega polynomial gives the total number of edges of the graph, and at *x* = 1, the Theta polynomials give the same result. The relations for the topological indices related to these polynomials are as follows:

1 $${\{{\rm{\Omega }}^{\prime} ({\rm{G}},{\rm{x}})\}}_{x=1}=\sum _{c}\,m\,c={\boldsymbol{\Omega }}({\boldsymbol{G}}){\boldsymbol{,}}$$

2 $${\{{\rm{\Theta }}^{\prime} ({\rm{G}},{\rm{x}})\}}_{x=1}=\sum _{c}\,m\,{c}^{2}={\rm{\Theta }}\,({\boldsymbol{G}}){\boldsymbol{,}}$$

3 $${\{{\rm{\Pi }}^{\prime} ({\rm{G}},{\rm{x}})\}}_{x=1}=\sum _{c}\,m\,c(e-c)={\boldsymbol{PI}}({\boldsymbol{G}}){\boldsymbol{,}}$$

4 $${\{sd^{\prime} ({\rm{G}},{\rm{x}})\}}_{x=1}=\sum _{c}\,m\,{c}^{2}(e-c)={\boldsymbol{sd}}({\boldsymbol{G}}).$$

The following Table 2. shows the Omega, Theta, PI, and Sadhana indices calculated from their related polynomials.

**Table 2:** Topological indices of the counting polynomials.

N	Ω(*G*)	Θ (*G*)	*PI*(*G*)	Sd(G)
1	31	79	882	372
2	48	178	2126	672
3	65	287	3938	1040
4	82	406	6318	1476
5	99	535	9266	1980

In^24^, John *et al*. proposed the following formulae to calculate the ***PI*** index in terms of the Omega and Theta indices by considering relations (1) and (2).

5 $${\boldsymbol{PI}}({\boldsymbol{G}})={\{{\boldsymbol{\Omega }}{\boldsymbol{^{\prime} }}{({\rm{G}},{\rm{x}})}_{{\boldsymbol{x}}=1}\}}^{2}-\{{\rm{\Theta }}^{\prime} {({\rm{G}},{\rm{x}})}_{{\boldsymbol{x}}=1}\}.$$

The omega index and the theta polynomial give the same result at *x* = 1; therefore, in terms of the theta polynomial relation **(5)** can be calculated as follows:

6 $${\boldsymbol{PI}}({\boldsymbol{G}})={\{{\rm{\Theta }}{({\rm{G}},{\rm{x}})}_{{\boldsymbol{x}}=1}\}}^{2}-\{{\rm{\Theta }}^{\prime} {({\rm{G}},{\rm{x}})}_{{\boldsymbol{x}}=1}\}.$$

The Sadhana index ***Sd*** (***G***) was defined by Khadikar *et al*. (for more details, we refer readers to^6,23,25,26^).

The Sadhana index is

7 $${\boldsymbol{Sd}}\,\,({\boldsymbol{G}})=\sum _{{\boldsymbol{c}}}\,{\boldsymbol{m}}({\boldsymbol{G}},{\boldsymbol{c}})(|{\boldsymbol{E}}({\boldsymbol{G}})|-{\boldsymbol{c}}),$$

where ***m*** (***G***, ***c***) is the number of strips of length *c*. The Sadhana polynomial *Sd* (*G*, *x*) was defined by Ashrafi *et al*. in 2008 (see^27^).

We proposed a new method to calculate the Sadhana index ***Sd*** (***G***) in terms of the Omega index and the Omega polynomial, as given below:

8 $${\boldsymbol{Sd}}\,\,({\boldsymbol{G}})=\{{\rm{\Omega }}^{\prime} ({\rm{G}},\,{{\rm{x}})}_{x=1}\}\{{\rm{\Omega }}{({\rm{G}},{\rm{x}})}_{x=1}-1\},$$

where ${\rm{\Omega }}^{\prime} {({\rm{G}},{\rm{x}})}_{x=1}$ is for the Omega index and Ω(*G*, *x*)_*x*=1_ is for the Omega polynomial at *x* = 1.

In Table 3, the PI and Sadhana indices are calculated with the help of the Omega and Theta polynomials.

**Table 3:** ***PI*** (***G***) and **Sd** (**G**) topological indices in terms of the Omega and Theta indices.

n	*PI* (*G*) = {Ω′ (G, x)_*x*=1_}^2^ − {Θ′(G, x)_*x*=1_}	*PI* (*G*) = {Θ(G, x)_*x*=1_}^2^ − {Θ′(G, x)_*x*=1_}	Sd (G) = Ω′ (G, x)_*x*=1_ {Ω (*G, x*))_*x*=1_ − 1}
1	882	882	372
2	2126	2126	672
3	3938	3938	1040
4	6318	6318	1476
5	9266	9266	1980

## Results

The Omega and Theta polynomials count the equidistant edges of the graph, while the Sadhana and PI polynomials count the nonequidistant edges of the graph. These polynomials help researchers discuss and predict the molecular structure without necessarily having to refer to quantum mechanics. Hence, we sum up this paper with the following results:

$${\boldsymbol{PI}}({\boldsymbol{G}})={\{{\rm{\Theta }}{({\rm{G}},{\rm{x}})}_{{\boldsymbol{x}}=1}\}}^{2}-\{{\rm{\Theta }}{({\rm{G}},{\rm{x}})}_{{\boldsymbol{x}}=1}\},$$

$${\boldsymbol{Sd}}\,({\boldsymbol{G}})\,=\,\{{\rm{\Omega }}^{\prime} {({\rm{G}},{\rm{x}})}_{{\boldsymbol{x}}=1}\}\{\Omega {(G,x)}_{{\boldsymbol{x}}=1}-1\}.$$
